# Wind damage dataset for buildings from 2016 tropical cyclone Winston in Fiji

**DOI:** 10.1016/j.dib.2025.111463

**Published:** 2025-03-13

**Authors:** Ryan Paulik, Shaun Williams, Misaeli Funaki, Richard Turner

**Affiliations:** a Meteorology and Remote Sensing, National Institute of Water and Atmospheric Research (NIWA), 301 Evans Bay, Greta Point, Wellington 6021, New Zealand; b Environmental Hazards, National Institute of Water and Atmospheric Research, 10 Kyle Street, Riccarton, Christchurch 8011, New Zealand; c Fiji Meteorological Service, Korowai Road, NAP 0351. Nadi Airport, Fiji

**Keywords:** Tropical cyclone Winston, Extreme winds, Building damage, Post-event survey, Vulnerability models

## Abstract

Extreme winds caused by tropical cyclones offer a unique opportunity to evaluate physical damage to building structures. On 20 February 2016, Category 5 Tropical Cyclone Winston (TC Winston) made landfall in Fiji, causing damage to over 30, 000 buildings. This article presents an empirical wind building damage dataset for Fiji collected from onsite damage assessments in the TC Winston aftermath. The dataset represents over 700 building-specific records of hazard, building and damage variables recorded during a four-day survey in March 2016. Physical damage to building structures, contents, stock, equipment and plant are presented, along with disruption to residential building habitability and non-residential building services. The dataset provides a valuable record of building damage caused by TC Winston extreme winds that can be used with numerical wind model hazard intensity outputs to formulate building-specific wind vulnerability models for damage prediction in future extreme wind events.

Specifications Table

**Table:** 

Subject	Engineering
Specific subject area	Post-event cyclonic wind building damage mapping to support predictive impact model development for extreme wind events.
Type of data	Table
Data collection	Building damage samples were recorded from onsite assessments over four days between 15–18 March 2016. A digital survey approach was used to record damage samples using the Real Time Asset Capture Tool (RiACT) , an Android-based for capturing and storing spatial information about building attributes and physical damage [1]. Global positioning system (GPS) functionality enabled accurate location positioning of wind-damaged buildings. The spatial information collected for each assessed building included GPS coordinates, evidence of direct wind damage and secondary hazards (i.e., water ingress), physical and non-physical building attributes and damage state.
Data source location	Institution: National Institute of Water and Atmospheric Research Town: Ba, Tavura, Rakiraki (Vaileka) , Volivoli, Nakorokula and Navolau Region: Viti Levu Country: Fiji
Data accessibility	Zenodo: [2]. https://zenodo.org/records/14353428 Data (CSV) : https://zenodo.org/records/14353428/files/2016TCWinston_DamageData.csv?download=1
Related research article	None

## 1 Value of the Data

• This dataset is the first publicly available collection of building-specific wind damage observations in Fiji for 2016 Tropical Cyclone Winston.
• The dataset contains 700 building damage samples, each containing detailed information on the evidence of direct wind damage and secondary hazards (i.e., water ingress) , physical and non-physical building attributes and damage state.
• The dataset can be used with numerical wind model hazard intensity outputs to formulate building-specific wind vulnerability models for damage prediction in future extreme wind events.
• The dataset can be integrated with analogous empirical data to develop a broader understanding of wind damage to building structures.

## 2 Background

On 20 February 2016, Category 5 Tropical Cyclone Winston (herein TC Winston) made landfall in Fiji. The cyclone's path across the north of Fiji caused the most extensive damage in Taveuni, Savusavu, Koro, Tavua, Rakiraki and Ba. A Government of Fiji-led post-disaster needs assessment reported that TC Winston caused 44 fatalities with more than 120 injured and a further 40,000 people requiring evacuation and shelter assistance [3]. In addition, more than 30,000 buildings were reported to be damaged or destroyed. TC Winston was an opportunity to collect empiical data on physical building damage caused by extreme wind events. Observations from the Fiji climate station network indicated that sustained winds (10-min average) experienced during the event exceeded 34 m/s in many areas with 3-s gusts potentially exceeding 70 m/s [4]. Extreme wind events of this magnitude are infrequent, and detailed information on wind hazard impacts on buildings is often not recorded. This data article presents a wind damage dataset for buildings collected after TC Winston from onsite surveys on Viti Levu island, Fiji.

## 3 Data Description

The wind damage dataset is structured by representing each building object identifier as rows (701), and its associated location, hazard, building and damage variables as columns (27). Building-specific variables are presented here in Table 1, forming a nominal-, ordinal- and interval-scaled data structure. The wind damage dataset is stored in a comma-separated value (.csv) file with latitude (X_coord) and longitude (Y_coord) coordinates identifying building locations for further spatial analyses using GIS software or open software programmes.

**Table 1:** Location, hazard, building and damage variables from TC Winston on Viti Levu island, Fiji.

Variable		Types or Description	Data Type	Unit or Value	Column Heading
Location	Object	Building object identifier	Integer	1 to ∞	ID
	Latitude	Latitude value in WGS84 (ESPG: 4328)	Decimal	Decimal degrees	Y_coord
	Longitude	Longitude value in WGS84 (ESPG: 4328)	Decimal	Decimal degrees	X_coord
Hazard	Shielding	‘Complete', ‘None', ‘Partial'	Text	3 classes	Shielding
	Water Ingress	Water ingress presence in building	Boolean	0 = false; 1 = true	Water_Ingress
Building	Attachment	Detached; Joined; Attached	Text	3 classes	Attachment
	Structural Frame	Brick masonry; Concrete masonry; Timber; Steel	Text	4 classes	Structural_Frame
	Foundation Type	Concrete Slab; Pile; Solid wall with Earth Fill	Text	4 classes	Foundation_Type
	Foundation Material	Concrete; Mixed; Timber	Text	3 classes	Foundation_Material
	First Storey Floor Height	First finished floor level height in meters above ground in meters	Decimal	m	FS_Floor_Height
	Second Storey Floor Height	Second finished floor level height in meters above ground in meters	Decimal	m	SS_Floor_Height
	Storeys	Number of complete building floor levels	Integer	1 to ∞	Storeys
	Condition	Good; Moderate; Poor	Text	8 classes	Condition
	Roof Cladding	‘Concrete; ‘Sheet Metal/Iron; ‘Corrugated Iron;‘Sheet Metal; ‘Metal Tile'	Text	5 classes	Roof Cladding
	Roof Pitch	Flat (0-2 degree) ; Near Flat (2-10 degree) ; Moderate (10-40 degree) ; Steep (greater than 40 degree)	Text	4 classes	Roof_Pitch
	Roof Type	‘Mono; ‘Hip; ‘Gable; ‘Complex'	Text	4 classes	Roof_Type
	Primary Use	Commercial: Accommodation; Commercial: Fast Moving Consumer Goods; Commercial: Office; Commercial: Retail; Community; Education; Government; Hospital, Clinic; Industrial: Manufacturing; Industrial: Storage; Industrial: Workshop; Religious; Residential	Text	13 classes	Primary_Use
	Wall Cladding	‘Concrete Masonry', ‘Corrugated Iron', ‘Open', ‘Plastic', ‘Sheet Metal', ‘Timber'	Text	6 classes	Wall_Cladding
	Proportion or wall openings	‘0-20 %', ‘20-40 %', ‘40 - 60 %', ‘40-60 %', ‘60-80 %', ‘80-100 %'	Text	6 classes	Wall_Openings
Damage	Damage State	‘DS0: None’, ‘DS1: Insignificant’, ‘DS2: Minor’, ‘DS3: Moderate’, ‘DS4: Severe’, ‘DS5: Complete’	Text	6 classes	Damage_State
	Contents Damage Ratio	‘0′, ‘0 - 0.1′, ‘0.1 - 0.5′, ‘0.5 - 0.8′, ‘0.8 - 1′	Text	5 classes	Contents_DR
	Equipment and Plant Damage Ratio	‘0’, ‘0 - 0.1’, ‘0.1 - 0.5’, ‘0.5 - 0.8’, ‘0.8 - 1’, ‘Not Applicable’	Text	6 classes	EquipmentPlant_DR
	Stock Damage Ratio	‘0’, ‘0 - 0.1’, ‘0.1 - 0.5’, ‘0.5 - 0.8’, ‘0.8 - 1’, ‘Not Applicable’	Text	6 classes	Stock_DR
	Residential Building Habitability	‘Habitable’, ‘Uninhabitable’, ‘Not Applicable’	Text	4 classes	Residential_Habitability
	Residential Building Disruption Time	Number of days normal residential building service is disrupted	Text	Days	Residential_Disruption_Days
	Non-residential Building Service Disruption	‘Disrupted’, ‘Operating’, ‘Not Applicable’	Text	Days	NonResidential_Disruption
	Non-residential Building Service Disruption Time	Number of days normal building service and business operations is disrupted	Text	Days	NonResidential_Disruption_Days

## 4 Experimental Design, Materials and Methods

**Fig. 1:**
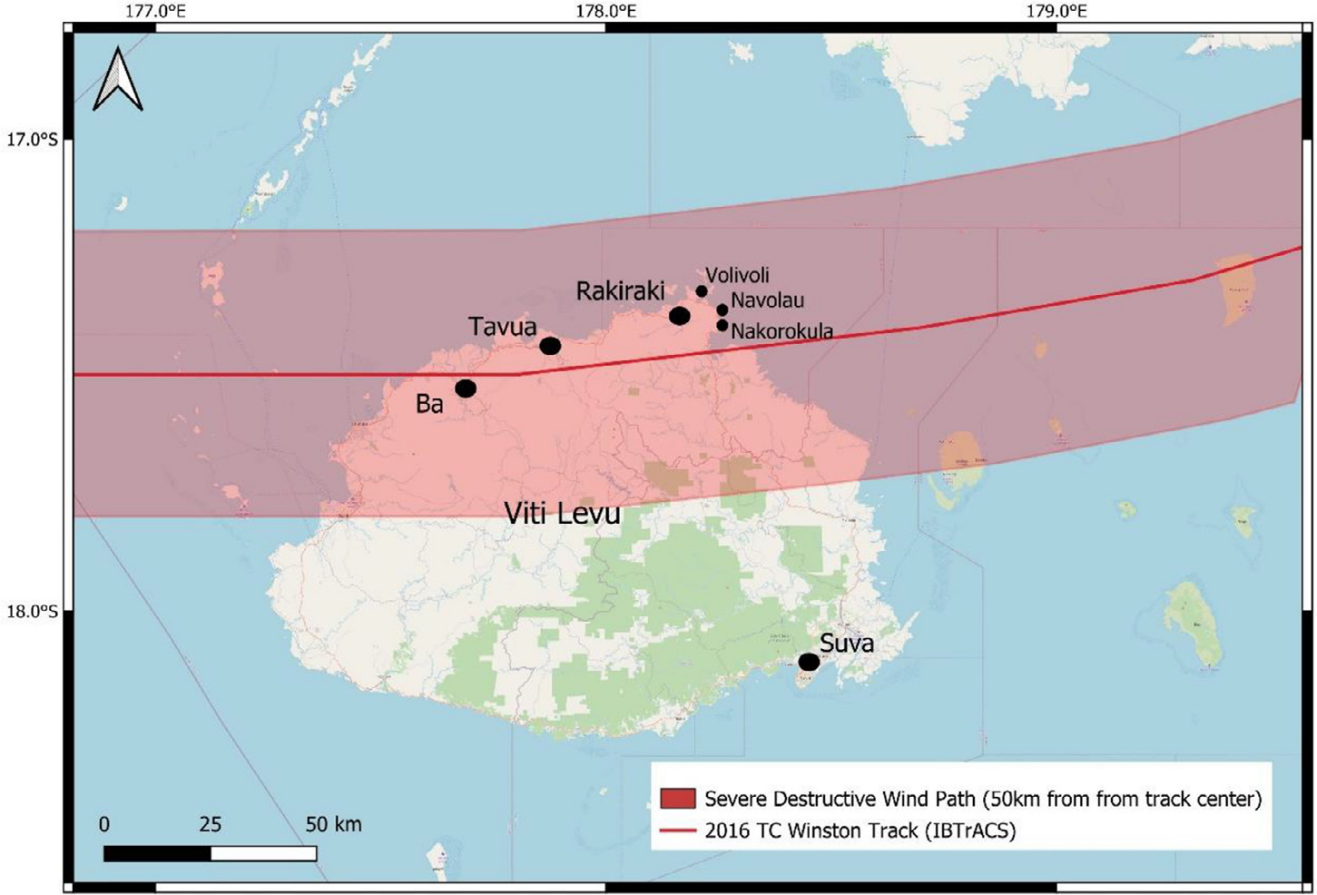
Wind-damaged building locations surveyed after 2016 TC Winston on Viti Levu, Fiji). Source: [5,6].

Wind damage data was collected onsite building assessments over a four-day period from 15–18 March 2016, three weeks after TC Winston made landfall in Fiji. The Fiji-New Zealand survey team (9 people) represented several agencies: Fiji Meteorological Service (2 people), Western Province Department of Works (2 people), Ministry of Rural, Maritime Development and National Disaster Management (1 person) National Institute of Water and Atmospheric Research (3 people) and GNS Science (1 person) . A pre-field deployment briefing was held at the Fiji Meteorological Service office on March 14^th,^ to identify primary locations for building damage assessments. Locations were selected due to their broad disparity in physical and non-physical building attributes and wind damage severity. Onsite building assessments for the first three days focused on the Rakiraki District in Fiji's Ra Province, northwest Viti Levu island. Rakiraki's Vaileka township and surrounding settlements were targeted for damage sample collection along with coastal settlements at Volivoli, Nakorokula and Navolau (Fig. 1). On the fourth day, onsite assessments focused on non-residential buildings (e.g., schools, churches) damaged in Ba and Tavua townships.

A digital survey was designed to record building attributes and damage states. Data samples were recorded using the Real Time Asset Capture Tool (RiACT), an Android based application with configurable templating to enter and store spatial information on building attributes and damage [1]. Global positioning system (GPS) functionality enabled accurate location positioning of damaged buildings.

Spatial information collected for buildings exposed to extreme winds included GPS coordinates, evidence of direct wind damage and secondary hazards (i.e., water ingress), physical and non-physical building attributes and damage state. Wind and rainfall hazard intensities could not be measured onsite. Water ingress damage observed onsite was recorded as Boolean values. Wind shielding afforded by nearby buildings was also recorded. Fourteen building attributes were recorded onsite, with physical attributes including: Attachment; Structural Frame, Foundation Type, Foundation Material, First Storey Floor Height, Second Storey Floor Height, Number of Storeys, Condition, Roof Cladding, Roof Design, Roof Pitch, Wall Cladding and Wall Openings. Primary building use was the only non-physical attribute recorded. Non-integer or decimal attribute values were pre-coded in RiACT resulting in a nominal-, ordinal- and interval-scaled data structure.

Building structure ‘wind damage states’ (DS) were categorised using an ordinal scale ranging from DS0 (No damage) to DS3 (Complete damage). The scheme's six-state damage division provided consistent damage descriptors across different building types. Damage states describe physical building damage observed onsite, indicating their potential for reinstatement to a pre-damaged condition (Table 2). The damage scheme derived a consistent damage database that could be integrated with field or remote sensed building damage datasets external to this survey.

**Table 2:** Damage state classification and descriptions for buildings surveyed after 2016 TC Winston.

Damage State (DS)	Damage State Classification	Damage Description	Condition of the structure
DS0	None	No damage to building structure	Promptly reusable without repair or cleaning
DS1	Insignificant	Slight damages to external non-structural components	Promptly reusable without urgent repair or cleaning
DS2	Minor	Some damage to roof (< 10 % removed) and/or internal non-structural components due to water ingress	Possible use only after urgent damage repair and cleaning
DS3	Moderate	Heavy damage to roof (10 % to 100 % removed) , major water ingress and damage to non-structural components. The structural integrity of external walls remains mostly intact.	Possible use after major damage repair and retrofitting of roof structure and non-structural components
DS4	Severe	Destructive damage to roof (>50 % removed) , walls and openings (<50 % of all walls) and/or columns (several bent or destroyed) . Major water ingress and damage to all non-structural components	Loss of functionality (system collapse) . Only repairable at significant repair and retrofitting cost
DS5	Complete	Blown away, only foundation remaining on footprint	Loss of functionality (system collapse) . Non-repairable or great cost for retrofitting

Non-structure building damage was recorded onsite for both residential and non-residential buildings. Non-structure damage relates to building contents, stock and equipment/plant. The difficulty in estimating proportional non-structural building item damage relative to their replacement cost (i.e., ‘cost of repair’/’cost of replacement’) onsite meant surveyors used ordinal values for damage ratio classification. Where possible, damage ratio estimates were derived based on advice and experiences from property owners or occupants, or in cases where advice was not available, surveyors estimated a damage ratio range based on damaged items at the building site or building structure damage observed (e.g., DS1 may translate to a content DR = 0 when a building sustains no internal damage) .

Habitability and business disruption were recorded for each building surveyed. Residential buildings were deemed either habitable or uninhabitable based on their current occupancy at the time of visit. Business owners or staff who were available at non-residential building sites were asked whether normal business activities could operate from the building after TC Winston and the total downtime (business days) for disruption to business activities.

## Limitations

Onsite damage surveys were three weeks after TC Winston made landfall in Fiji. Wind hazard and rainfall intensities could not be measured onsite with the presence of water ingress damage recorded as Boolean values (Table 1). Where residential buildings were uninhabitable or non-residential building services were disrupted at the time of onsite surveys, the total number of uninhabitable or disruption days was recorded as ‘Unknown’.

## Ethics Statement

The authors have read and follow the ethical requirements for publication in Data in Brief. The current dataset does not involve human subjects, animal experiments, or data collected from social media platforms.

## CRediT authorship contribution statement

**Ryan Paulik:** Conceptualization, Methodology, Validation, Formal analysis, Investigation, Data curation, Writing – original draft, Funding acquisition. **Shaun Williams:** Conceptualization, Methodology, Investigation, Writing – original draft. **Misaeli Funaki:** Investigation, Resources, Project administration. **Richard Turner:** Conceptualization, Investigation, Project administration.

## Data Availability

ZenodoWind Damage Dataset for Buildings from 2016 Tropical Cyclone Winston in Fiji (Original data).
